# Response to "optic nerve sheath diameter guided detection of sepsis associated encephalopathy"

**DOI:** 10.1186/s13054-020-03395-3

**Published:** 2020-12-07

**Authors:** Ziyue Yang, Tongwen Sun

**Affiliations:** General ICU, The First Affiliated Hospital of Zhengzhou University, Henan Key Laboratory of Critical Care Medicine, Zhengzhou Key Laboratory of Sepsis, Zhengzhou, 450052 China

**To the Editor:**

Dear Professor Suresh,

Thanks for your interest in our research.

First, regarding the blind method, being uninformed of the clinical diagnoses of patients is unavailing, since clues will still be found in patients’ clinical manifestations (soberness/delirium/irritability/sleepiness/coma). Therefore, two trained physicians in intensive ultrasound were selected for joint measurement. After each measurement, the images were stored on the ultrasound machine, on which the optic nerve sheath diameter (ONSD) was measured. This can minimize the influence of subjective factors on the measurement. This is perceived as an appropriate blind method [1].

Second, the sepsis patients included in the research were aged between 18 and 93. However, in our experiment, we did not encounter patients with split or trabecular optic nerve sheaths, nor difficulties in the measurement due to skeletal problems, though these did appear in clinical work. Therefore, more comprehensive consideration is necessary during research on the large-sized sample. Any of these situations will be excluded if it has a big impact on the result.

Third, no significant difference was identified in serum albumin concentration among patients in different groups (sepsis group, sepsis-associated encephalopathy group, and sepsis-associated encephalopathy recovery group) (28.9 [25.5,31.05], 30.5 [27.6,32.2], 34.75 [29.7,36]), especially between the sepsis group and the sepsis-associated encephalopathy group. Therefore, a small correlation between albumin and ONSD cannot prove the role of albumin in dominating intracranial pressure/onsd. Furthermore, intracranial pressure may also be affected by factors such as albumin, blood sugar, bedside angle, ventilator parameters, end-tidal carbon dioxide concentration, blood pressure, respiratory rate, and state of consciousness [2, 3]. The results will be further revealed in follow-up research.

Finally, thanks again for your letter.

## Data Availability

All data generated or analyzed during this study are included in this published article and its supplementary information files.
